# Bacterial communities associated to Chilean altiplanic native plants from the Andean grasslands soils

**DOI:** 10.1038/s41598-018-37776-0

**Published:** 2019-01-31

**Authors:** Beatriz Fernández-Gómez, Jonathan Maldonado, Dinka Mandakovic, Alexis Gaete, Rodrigo A. Gutiérrez, Alejandro Maass, Veronica Cambiazo, Mauricio González

**Affiliations:** 1 FONDAP Center for Genome Regulation, Santiago, Chile; 2 Department of Bioinformatics and Genetic Expression, Institute of Nutrition and Food Technology, Universidad de Chile, El Líbano 5524, Macul, Santiago, Chile; 3 Department of Molecular Genetics and Microbiology, Pontificia Universidad Católica de Chile, Av. Libertador Bernardo O’Higgins 340, Santiago, Chile; 4 Millennium Institute for Integrative Plant Systems and Synthetic Biology, Pontificia Universidad Católica de Chile, Av. Libertador Bernardo O’Higgins 340, Santiago, Chile; 5 Mathomics, Center for Mathematical Modeling, Universidad de Chile, Beauchef 850, Santiago, Chile; 6 Department of Mathematical Engineering, Universidad de Chile, Beauchef 850, Santiago, Chile

## Abstract

The rhizosphere is considered the primary place for soil microbiome differentiation and plays a key role in plant survival, especially for those subjected to environmental stress. Using high-throughput sequencing of the 16S rRNA gene, we analyzed and compared soil bacterial communities associated to four of the most abundant high altitude native plant species of the Chilean Andean grasslands. We examined three soil compartments: the rhizosphere (bacteria firmly attached to the roots), the rhizosphere-surrounding soil (bacteria loosely attached to the roots) and the bulk soil (plant-free soil). The rhizosphere microbiome was in all cases the least diverse, exposing that the bulk soil was a more complex environment. Taxonomic analysis revealed an abrupt change between the rhizosphere and the rest of the non-rhizospheric soils. Thus, while rhizobacterial communities were enriched in Proteobacteria (mainly Alphaproteobacteria), Actinobacteria (mostly Blastocatellia) dominated in bulk soils. Finally, we detected certain taxonomic rhizosphere signatures, which could be attributed to a particular genotype. Overall, our results indicate that the thin layer of soil surrounding the roots constitute a distinctive soil environment. This study contributes to expand the knowledge about soil bacterial communities in the Chilean highlands and takes the first step to understand the processes that might lead to the rhizosphere differentiation in that area.

## Introduction

Soils constitute an extraordinarily diverse microbial environment^1,2^. In the rhizosphere, plants interact with soil microorganisms developing their own microbial community or “microbiome”^3^. Root exudates are known to influence rhizobacterial community differentiation^4–8^ by either stimulating or repressing bacterial growth^9,10^ or by altering the soil microhabitat^11–13^. Soil type and plant genotype has been defined as the major drivers in shaping rhizobacterial communities^14–18^. Other biotic and abiotic factors that shape bacterial diversity and abundance in the rhizosphere are the developmental stage of the plant, microbial interactions, biogeography, soil pH, and carbon content^19–26^.

The importance of rhizobacteria improving plant health and growth^27–29^ has led to a widespread interest for studying the rhizosphere of many agroecosystems, mainly crops^30–37^. However, studies of soil bacterial communities associated with native vegetation are more scarce^18,23,38–40^, especially in extreme environments such as the Andean grasslands^20,41,42^. The Andean grasslands or “steppe” corresponds to the upper limit for plant life in the Atacama Desert. It constitutes the highest (>3,500 meters above the sea level; m.a.s.l.) and the harshest biogeographic region in the Altiplano^43^. However, it surprisingly supports a wide variety of plant species with clear dominance of perennial grasses^44–48^. In that extreme environment, grasslands play an important role contributing to the stabilization of soils, increasing nutrient availability and water-holding capacity, and avoiding soil erosion^49^. The ability of plants to adapt and survive to extreme conditions depends on their association with a specific rhizosphere microbiome^50–52^. The study of the rhizosphere in steppe plants is crucial to understand their ability to tolerate high levels of abiotic stress typical of arid areas such as high salinity, extreme temperature oscillation, high UV intensity or low nutrient availability^53^.

Microbial communities in Atacama soils are well-known, mostly because this habitat is considered the dry edge for life^54–59^ and it has always intrigued scientists for its similarities with Mars soils^60^. However, little is known about the diversity of bacterial communities associated to plants in the Atacama highlands^40^. In this study, carried out in a location above 3,800 m.a.s.l., we aimed to describe and compare soil bacterial communities associated to grasslands native plants in an attempt to identify potential groups of bacteria selected and enriched in the rhizosphere, together with potential factors that could influence or control the structure of bacterial communities in this type of soil. Our findings indicated that some plants exerted a selective pressure on bacterial communities promoting the enrichment of Alphaproteobacteria in the rhizosphere, a class known for including plant-growth promoting rhizobacteria (PGPR). Although more extensive studies are needed to fully comprehend de diversity and distribution of rhizobacterial communities in the Chilean Altiplano and to elucidate the environmental factors that regulate that diversity, this work sets the baseline for this kind of studies in this extreme environment.

## Results

### Area, plants, and soil characteristics

**Figure 1:**
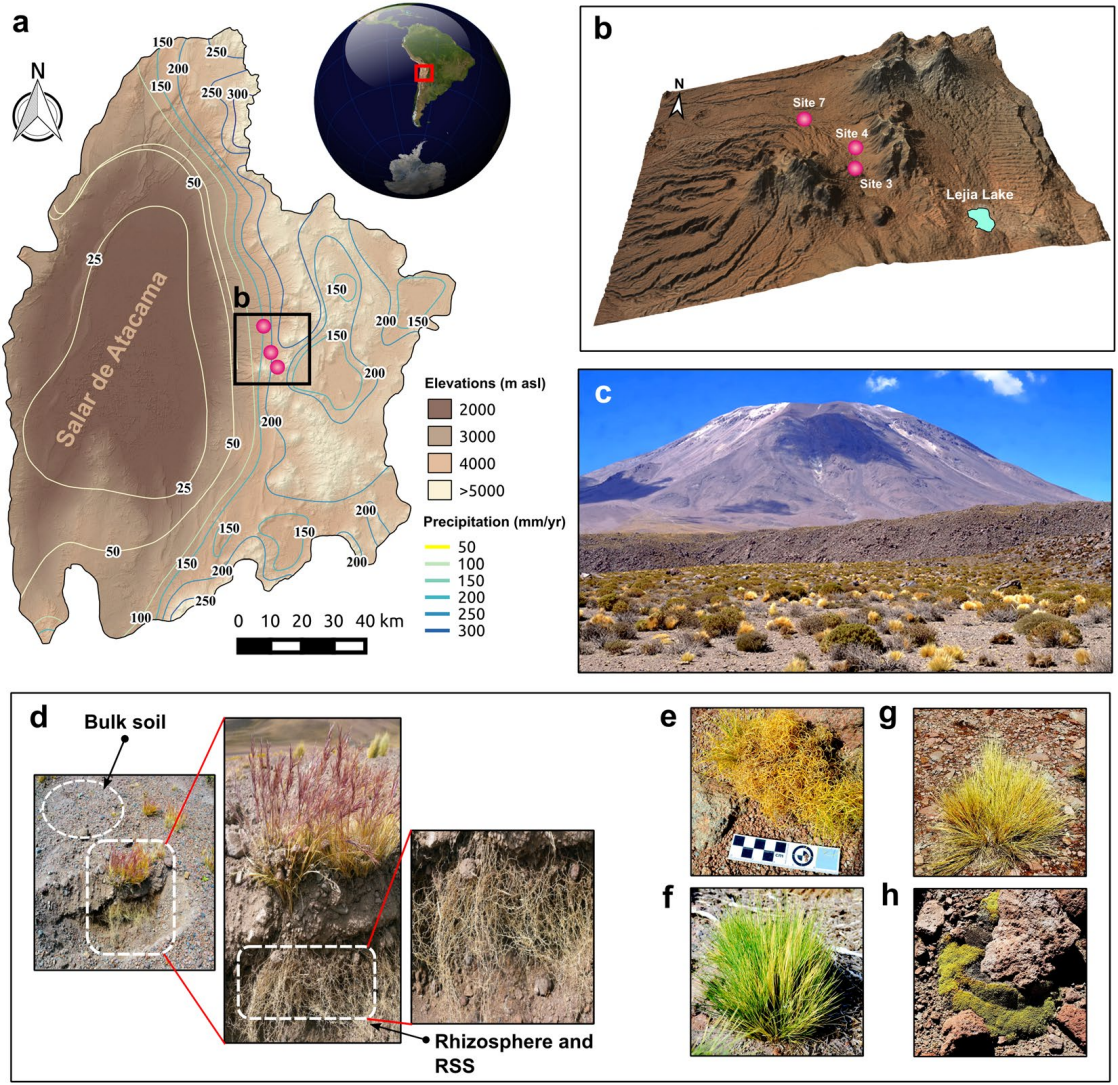
Sampling site and plants investigated. Regional context of the study site in northern Chile showing (**a**) the location of the Salar de Atacama and adjacent Andes and (**b**) a digital elevation model indicating the sampling sites (colored dots). (**c**) Picture of the Andean steppe in the Chilean Altiplano, representative of sites 3, 4, and 7. (**d**) The three compartments sampled in this study. (**e–h**) The four plant species used in this study: *Calamagrostis crispa*, *Nassella nardoides*, *Jarava frigida*, and *Pycnophyllum bryoides*. The software used to create the map was QGIS 2.18 with STRM30^106,107^ elevation model (Data: SIO, NOAA, U.S. Navy, NGA, GEBCO) and Landsat 8 Satellite image by the Operation Land Imager (OLI) (Data available from the U.S. Geological Survey).

The sampling area (Fig. 1a–c) was located between 3,870–4,270 m.a.s.l. (Table S1), corresponding to the Andean steppe of the Atacama Desert, northern Chile. This area receives more annual rainfall (100–200 mm) than the lower parts of the Atacama Desert (12–16 mm)^46,61^. We analyzed four plants species, three from the Poaceae family (Fig. 1d–h): *Calamagrostis crispa* (CAL, site 3), *Nassella nardoides* (NAS, site 3) and *Jarava frigida* (JAR, site 7); and a cushion-like herb from the Caryophyllaceae family (Fig. 1f): *Pycnophyllum bryoides* (PYC, site 4). These plants were chosen because they represented larger coverage percentages in the Andean steppe than other plant species. We defined three compartments or niches according to their proximity to the roots: the rhizosphere (bacteria firmly attached to the roots), the rhizosphere-surrounding soil (bacteria loosely attached to the roots; hereinafter RSS), and the bulk soil (plant-free soil) (Fig. 1d).

In order to perform a general description of the bulk soils, we analyzed their physicochemical structure and composition, as well as their soluble fraction composition (Table S1). Low values of pH, electrical conductivity (EC), and organic matter (OM) were common to all soils, as well as a high percentage of sand (close to 90%), which is typical of desert soils. Analysis of the soluble fraction showed that nitrogen (N), potassium (K), sodium (Na), calcium (Ca), and iron (Fe) were the predominant elements, whereas bromine (Br), barium (Ba), copper (Cu), and arsenic (As) were among the scarcest. We did not detect significant differences (Mann-Whitney U test; p < 0.05) among bulk soils, except for some nutrients such as As, Ca, Fe, K, and P between bulk soils of plants *Calamagrostis crispa* and *Nassella nardoides* (sampled in site 3); and *Jarava frigida* (sampled in site 7) (Table S1). Nevertheless, the sampling area seemed to be very homogeneous according to the bulk soils properties analyzed.

### Structure of the bacterial communities in the three soil compartments

Analysis of the raw sequence data yielded 3,648,675 reads after quality trimming. The total number of Operational Taxonomic Units (OTUs) obtained considering all samples (n = 36) was 10,810 (Table S2), as defined by 97% of similarity. Separated by compartment, 7,819 OTUs were identified in bulk soil samples, 8,244 OTUs in the RSS samples, and only 5,917 were detected in the rhizospheric samples (Table S2).

The vast majority of the OTUs found in the three compartments belonged to the rare biosphere, that is, they exhibited low relative abundances (<0.1%), while just a few OTUs exhibited high abundances (>10%) (Table S2).

**Figure 2:**
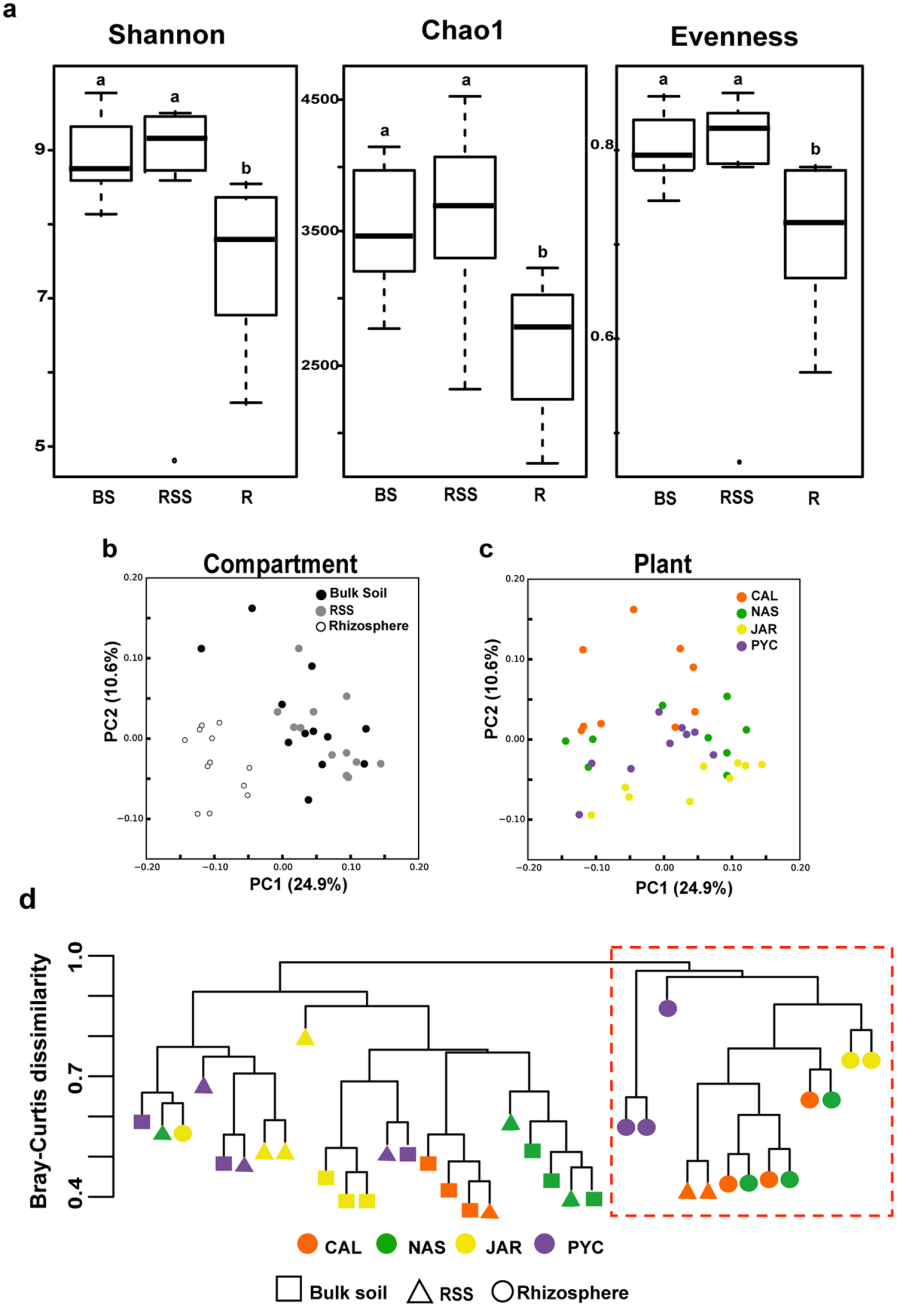
Structure and taxonomic composition of bacterial communities in the three soil compartments. (**a**) Alpha-diversity indicates a decreasing gradient in bacterial diversity from the bulk soil to the rhizosphere. Horizontal bars within boxes represent median. The tops and bottoms of boxes represent 75th and 25th quartiles, respectively. All outliers are plotted as individual points. BS: Bulk soil; RSS: rhizosphere-surrounding soil; R: Rhizosphere. ^a,b^Bars with different letters indicate statistically significant differences (Dunn’s test). (**b**) Principal coordinates analysis (PCoA) of bacterial communities based on the unweighted UniFrac distance matrix, as affected by soil compartment, and (**c**) plant species. (**d**) Bray-Curtis dissimilarity using all OTUs relative abundances present in all samples. The red square points out the cluster formed almost exclusively by rhizospheric samples.

Rhizospheric bacterial communities showed lower values of diversity (Shannon’s index H’), richness (Chao1 index), and evenness (Shannon’s equitability index) indices as compared to the RSS and the bulk soil (Fig. 2a and Table S3). Statistical analysis including the four plants showed significant differences among compartments (Kruskal-Wallis analysis of variance; p < 0.05) in terms of diversity (p = 0.00015), richness (p = 0.003), and evenness (p = 0.0005). Additional pairwise Dunn’s test analysis (Table S4) revealed a significant reduction in alpha-diversity in the rhizosphere compared to the RSS and the bulk soil (Table S4).

Principal coordinates analyses (PCoA) of the entire soil bacterial communities (36 samples) were performed to analyze the variation in bacterial abundance and taxonomic composition as affected by compartment and plant species (Fig. 2b,c). These plots revealed that the clearest separation between the rhizosphere and the rest of the surrounding soil samples was given by the variable “compartments” (Fig. 2b). However, PC1 and PC2 only explained 24.9% and 10.6% of total variance respectively, implying the existence of other factors that could affect grasslands soils bacterial communities. An additional hierarchical cluster based on the Bray-Curtis distance analysis (Fig. 2d) was performed to visualize differences in taxonomic composition between the rhizosphere and the other two soil compartments. A cluster containing most of the rhizosphere samples (red dashed square in Fig. 2d) appeared separated from a second principal cluster containing mainly RSS and bulk soil samples. We also observed that within the rhizospheric cluster (red dashed square in Fig. 2d), PYC rhizospheric samples (purple circles) appeared separated from the Poaceae samples (orange, green, and yellow circles), revealing differences in community composition at OTU level between Poaceae and Caryophyllaceae. Among the Poaceae, CAL and NAS revealed major taxonomic similarities in their rhizobacterial communities.

For each plant species, we determined the number of OTUs (relative abundance >0.03%) shared across all three compartments, which constituted what we called the plant core microbiome (Fig. S1). We observed that around 56% of all OTUs (64% in CAL, 45.4% in NAS, 57% in JAR, and 52.3% in PYC) present in the three compartments constituted the core microbiome. A substantial number of OTUs was also shared between the bulk soil and the RSS, and also between the RSS and the rhizosphere (Fig. S1). Thus, the RSS bacterial community appeared to be a subset of the bulk soil and, in turn, the rhizobacterial community a subset of the RSS. Actually, each compartment only exhibited an average of 3.7% of exclusive or non-core OTUs, that is, OTUs not found in any other compartment. Besides, the rhizosphere presented the highest percentage of non-core OTUs, with an average of 6%.

Analysis of the number of OTUs integrating the core microbiome of all plants together revealed that 358 OTUs (3.3%) were common to the four plants (Table S2). On the other hand, 457 OTUs (4.2%) were common to the three Poaceae (Table S2). Orders Rhizobiales, Sphingomonadales (both Alphaproteobacteria), Burkholderiales (Betaprotebacteria), and RB41 (Blatocatellia) were the most recurrent in the plant core microbiomes.

### Taxonomic composition of bacterial communities in the three soil compartments

**Figure 3:**
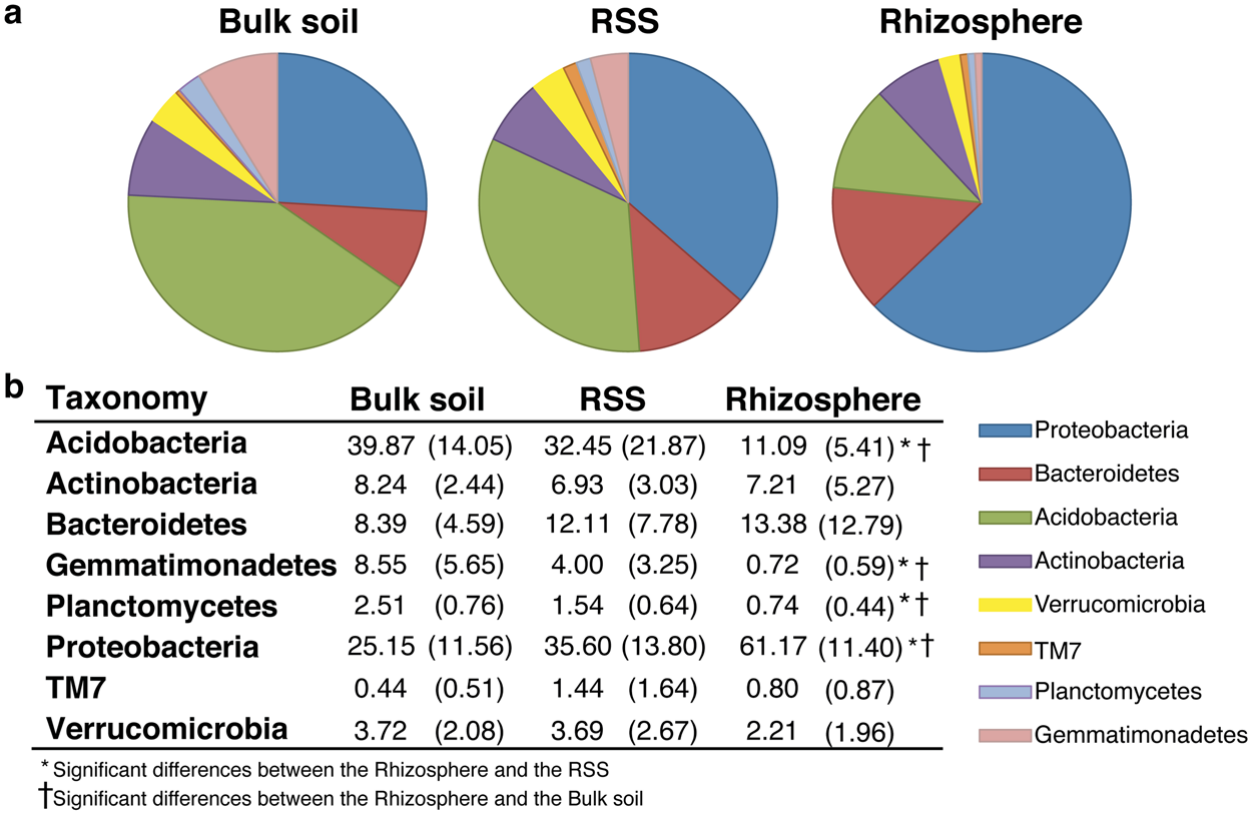
Taxonomic composition at phylum level and relative abundances (average per compartment; >1% in at least one compartment) in the three soil compartments for the three Poaceae. (**a**) Graphical representation and (**b**) table showing the actual data. Significant differences were calculated between phyla in the rhizosphere with respect to the RSS and with respect to the bulk soil. Numbers in brackets represent the standard deviations. RSS: rhizosphere-surrounding soil.

**Figure 4:**
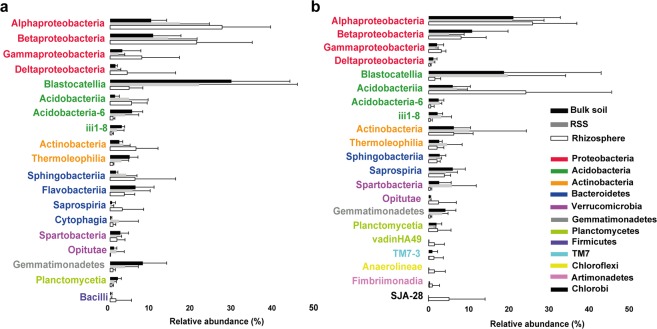
Taxonomic composition and relative abundances in the three soil compartments. Bars illustrate the mean relative abundance (average per compartment; >1% in at least one compartment) of the dominant classes ± standard deviation for (**a**) Poaceae and (**b**) *Pycnophyllum bryoides*.

To identify differences in the proportions of the relevant taxonomic groups across the three compartments, taxonomic sorting of the OTU sequences at phylum and class levels was performed (Figs 3, 4 and S2). Overall, the rhizosphere of the three species of Poaceae was dominated by the phylum Proteobacteria (Fig. 3) that mainly comprised Alpha- and Betaproteobacteria (28% and 21.5%, respectively) (Fig. 4a). In contrast, Acidobacteria prevailed in the bulk soil (40%) (Fig. 3) and was mostly represented by class Blastocatellia (30%) (Fig. 4a). In RSS, Proteobacteria and Acidobacteria were present in similar proportions (35.6% and 32.5%, respectively) (Fig. 3), close to the proportions found in the bulk soil. Both Proteobacteria and Acidobacteria showed remarkable differences in abundance between the rhizosphere and the rest of the soil (Fig. 4b). Bacteroidetes, Actinobacteria, and Verrucomicrobia did not show significant differences in relative abundance in the rhizosphere with respect to other soil compartments, but we observed a predilection of classes for a specific compartment. For example, Bacteroidetes in the rhizosphere were mainly represented by Sphingobacteriia (6%), whereas in the RSS and the bulk soil, class Saprospiria dominated (6%) (Fig. 4a). Likewise, class Actinobacteria was mainly represented in the rhizosphere (6.5%), while Thermoleophila (4.5%) prevailed in the bulk soil (Fig. 4a). Phylum Gemmatimonadetes was poorly represented in the rhizosphere (<1%); however, its abundance increased in the RSS and the bulk soil (4% and 8.5%, respectively) (Fig. 3). Significant differences (p < 0.05) in relative abundance among the main phyla and compartments in grasses are shown in Table S5. The stronger significant differences were detected between the rhizosphere and the other two compartments, specially the bulk soil. Interspecific differences in the taxonomic profile were observed among the three Poaceae (Fig. S3).

On the other hand, the cushion-like PYC exhibited a different pattern (Figs 4b and S2). Proteobacteria, Acidobacteria, and Actinobacteria were the most abundant groups in all three compartments with no significant differences observed between the rhizosphere and the rest of soil compartments (Fig. S2). Unlike the Poaceae, the rhizosphere of PYC was dominated by classes Alphaproteobacteria (26%) and Acidobacteriia (24%) (Fig. 4b). Besides, PYC exhibited in the rhizosphere more diversity in the less abundant phyla (TM7, Chloroflexi, Artimonadetes, Chlorobi) (Fig. 4b). Contrary to what was observed for the Poaceae species, PYC did not show significant differences (p < 0.05) in relative abundance of any of the main phyla among compartments (Table S5).

**Figure 5:**
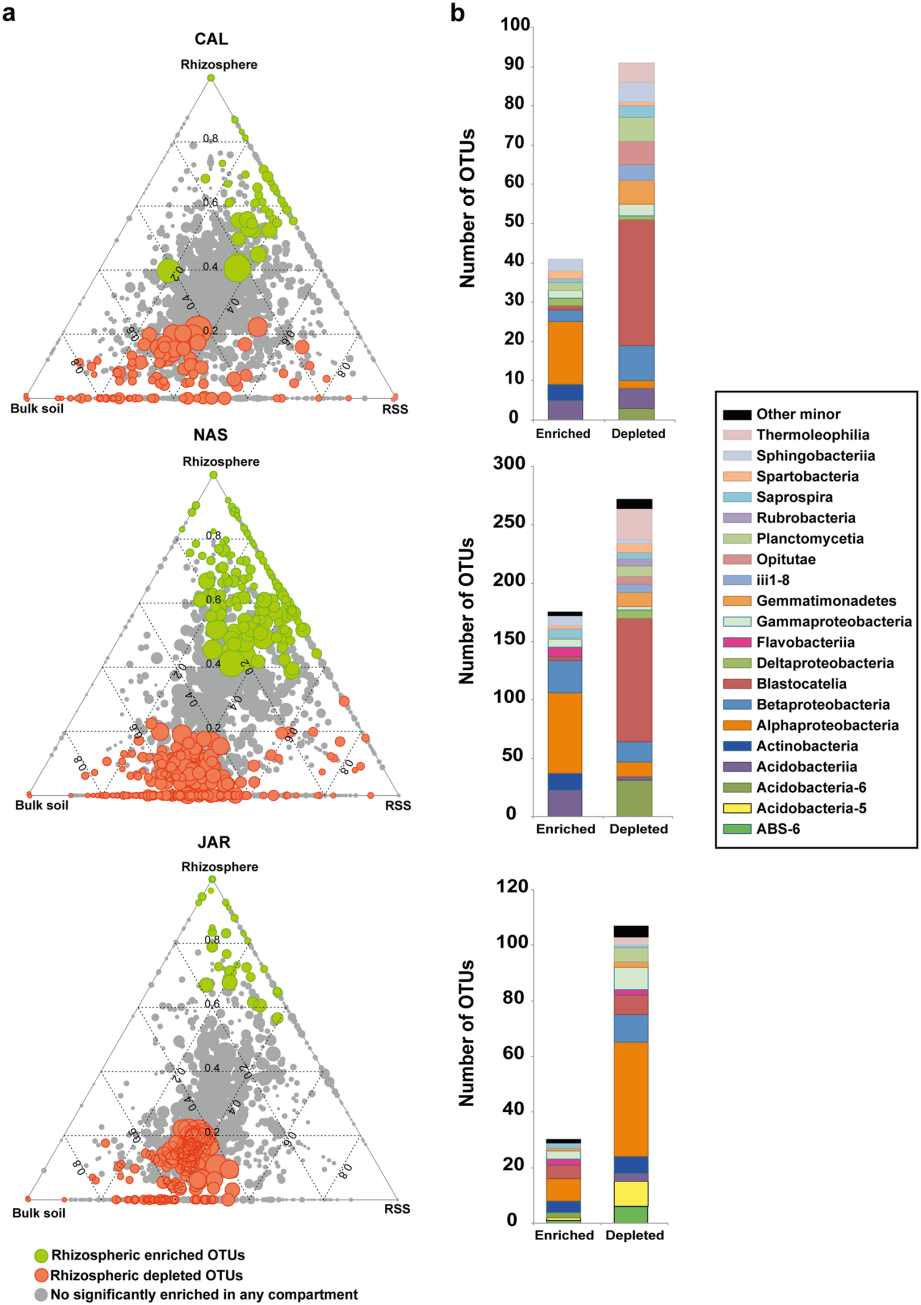
OTU enrichment and depletion in the rhizosphere. (**a**) Ternary plot representing all OTUs in the data set with relative abundance >0.03% in at least one sample (~80% of the total abundance). Dot size represents its mean relative abundance (weighted average) and their position is determined by the contribution of each compartment to its total relative abundance. The dotted grid and numbers inside the triangle indicate 20% increments of contribution from each compartment. Green dots represent OTUs significantly enriched in the rhizosphere. Orange dots represent OTUs significantly depleted in the rhizosphere (FRD; p < 0.05 in both cases). Gray dots represent OTUs not significantly enriched or depleted. (**b**) Histograms indicating the taxonomy (class level) of all enriched and depleted OTUs. Only those classes with at least three OTUs in one category (enriched or depleted) are represented. CAL: *Calamagrostis crispa*, NAS: *Nassella nardoides*, JAR: *Jarava frigida*, PYC: *Pycnophyllum bryoides*.

To identify the OTUs that correlated with community differentiation among compartments, we employed a differential OTU abundance analysis by fitting linear model analysis (see methodology) to detect the OTUs significantly enriched and depleted in the rhizosphere (Fig. 5a; green dots and orange dots, respectively). We observed that the vast majority of the OTUs were shared among the three compartments with no significant changes in their abundances (Fig. 5a; gray dots). Remarkably, PYC did not exhibit enrichment or depletion in any compartment. In general, all rhizospheric samples were depleted in OTUs (Fig. 5), that is, the RSS and the bulk soil were more enriched in OTUs than the rhizosphere. By plant especies, NAS exhibited the greater number of enriched and depleted OTUs (179 and 281, respectively) (Figs 5 and S2a,b). Besides, CAL and NAS shared a large number of enriched and depleted OTUs, but they just shared a few OTUs with JAR (Fig. S2a,b). Taxonomic variations between the enriched and depleted rhizospheric OTUs were also identified (Fig. 5b and Table S6). On average, Alphaproteobacteria dominated among the enriched OTUs in the Poaceae, while Acidobacteria (Blastocatellia) prevailed among the depleted OTUs.

Finally, we investigated the OTUs that for each Poaceae plant were enriched and, at the same time, were exclusive to the rhizosphere (not present in other compartments) (Fig. S4c and Table S6). Interestingly, the fraction of rhizospheric enriched and exclusive OTUs was plant species-specific, suggesting that each plant species selects or recruits a particular group of potentially beneficial OTUs. Genera *Bosea*, *Pseudomonas*, *Pedobacter*, *Kaistobacter*, and *Chyseobacterium* were detected among the most recurrent rhizospheric enriched OTUs (Table S7).

## Discussion

In this study, we assessed the changes in soil bacterial community structure and composition from an area corresponding to the steppe vegetation belt or grassland included in the altitudinal Talabre-Lejía transect^46^, across three different compartments defined according to their distance to plant roots. Taking into account that we already observed bacterial taxonomic changes driven by a combination of pH and nutritional factors^62^, here we aimed to describe and compare soil bacterial communities associated to native plants in an attempt to identify potential groups of bacteria selected and enriched in the rhizosphere, together with potential factors that could influence or control the structure of bacterial communities in this type of soil. Our results support the concept that, in addition to abiotic drivers (pH, nutrients), plants actively contribute to shape their rhizosphere microbiome. Thus, the roots would gradually change the original bacterial community found in the bulk soil and develop a rhizosphere community different from the ones present in the soil further away^63,64^.

Many studies have investigated other root compartments such as the rhizoplane (immediate surface root) or the endosphere (inside the root)^30,65^, but studies on what we named here the RSS are very scarce^23^. We believe that the incorporation of this compartment is crucial to estimate whether the differences in structure and composition between the rhizosphere and the bulk soil were due to gradual alterations through the RSS or, on the contrary, the result of abrupt changes between the rhizosphere and the rest of the surrounding soil.

Despite the small number of samples, we observed that, for a given plant, the three compartments shared a common bacterial community (core microbiome) (Fig. S1). That fact also indicated that the microbiome found in the RSS and the rhizosphere came from the same original bacterial community found in the bulk soil that, in turn, was determined by the environmental factors affecting these soils over time^66^. The exclusive OTUs found in the rhizosphere (non-core) could have arisen as a consequence of the difficulty in detecting them in the bulk soil by insufficient read depth or due to their low abundance^67–69^, nonetheless were enriched in the rhizosphere. Or, on the other hand, they could have been present in a “dormant stage” (e.g. spore-forming bacteria), which is typical in extreme environmental conditions^70,71^.

Analysis of the alpha-diversity revealed that the rhizosphere showed lower values of richness (Chao1) and diversity (Shannon’s) indices than the RSS and the bulk soil, suggesting that the non-rhizospheric bacterial communities were more complex than those in the rhizosphere. That fact has been evidenced in many other studies from different environments^8,26,31,72,73^, supporting the idea that a selective change in bacterial communities occurs within the first millimeters from the root system^6,12^. Taxonomic analyses also detected differences (in composition and relative abundances) in the rhizosphere with respect to the rest of the surrounding soil^27,66^. In our case, and only considering the Poaceae, the bulk soil was richer in Acidobacteria, while the rhizosphere was richer in Proteobacteria (Fig. 3). Our results agree with other studies^28,64^, including those in arid environments^20,22,23,40,41^. According to this, the Chilean Andean steppe could be comparable to other environments at phylum level. Nevertheless, as mentioned in other studies, it is at the lower taxonomical levels (genus or species)^74^ or in the less abundant taxa (rare biosphere)^75^ that the differences could be more evident. For example, Gammaproteobacteria was the most abundant class in the rhizosphere of plants in areas close to the Atacama Desert^40^ as revealed by DGGE and 454 pyrosequencing; however, in our samples, Alphaproteobacteria dominated the rhizosphere of both Poaceae and Caryophyllaceae. Furthermore, most of the enriched bacteria in the rhizosphere of the Poaceae belonged to the class Alphaproteobacteria (Table S6). Precisely, Alphaproteobacteria contains plant growth-promoting rhizobacteria (PGPR)^76–78^, suggesting that the plant could favor the growth of some members of this bacterial class. Besides, the alphaproteobacterial genus *Kaistobacter*, which was abundant and enriched in the rhizosphere of some of our plants (Tables S2 and S7), it has also recently been identified as a plant-disease suppressor^79^. In addition, of particular interest in the rhizosphere are the growth-promoting bacteria capable of fixing nitrogen. Some of the enriched OTUs in the rhizosphere of our Poaceae belonged to the genera *Bosea* (fam. Bradyrhizobiaceae) and *Pseudomonas*, both including nitrogen fixers^80^. Genus *Bradyrhizobium*, although not enriched in any rhizospheric sample, represented a substantial fraction in the Poaceae rhizosphere (on average 3%). In the case of PYC, the plant perhaps relies on another kind of plant growth-promoting bacteria since we found low percentages of potentially nitrogen fixers.

Gemmatimonadetes and Planctomycetes showed low abundance in the bulk soil and practically disappeared in the rhizosphere. Both groups were reported to be very abundant in the hyperarid soils at lower areas of the Atacama Desert (absolute desert)^55^, suggesting that these groups could have been affected by the changing conditions experienced by the bulk soils with the altitude, such as pH or moisture. Moreover, the microenvironment (root exudates) in the rhizosphere of the four plants analyzed might have not been favorable for these phyla, or they are simply not as beneficial as other groups to be recruited. Finally, members of Firmicutes, which have been found in high numbers in the rhizosphere of arid plants^52,81,82^, and some strains have been described plant-disease suppressor^83^, were found in low abundance in our rhizospheric samples. Crits-Christoph and collaborators^58^ also found low abundance of Firmicutes in the unplanted soils of the lower parts of the Atacama Desert. They indicated that this low abundance was due to the fact that most bacteria were in form of vegetative cells. Precisely, the formation of heat-resistant endospores by Firmicutes members is a well-known feature of this phylum^84^. In fact, the ability to produce spores would allow them to survive the environmental challenges of the desert, such as heat, desiccation and/or UV irradiation^85^.

The ternary plots revealed that the vast majority of the OTUs found associated to each plant species were ubiquitous, that is, they were found in the three soil compartments (Fig. 5; gray dots). This might be explained by the presence of generalists, which are well-adapted bacteria to diverse habitats by their capability to metabolize a large diversity of substrates^86,87^. It could also be explained by bacteria-bacteria interactions, and for the fact that the co-occurrence of certain types of bacteria might guarantee their success in all types of compartments^62^. Surprisingly, the rhizosphere turned out to be less enriched in OTUs than the non-rhizospheric soil, or not enriched at all as in the case of PYC. This result suggests that maybe only a few members of the rhizosphere could find the optimal conditions to thrive in this compartment, but also that these native plants only recruited a few OTUs that might be needed for their survival.

We detected interspecific variability within compartments (Fig. S3)^15^ that is, differences in the bacterial communities from a certain compartment, according to the plant genotype. Although growing in a very similar area and soil, all plants showed different bacterial compositions when comparing rhizosphere, RSS, and bulk soil bacterial composition. Besides, grasses species (CAL, NAS, and JAR) shared more taxonomic similarities among them than with the non-grass PYC (Fig. 2e). Although further investigations should be done, including the addition of more samples, we think that these differences could be due to the fact that, as observed in several studies, plant genotype (even among different types of the same plant species) is able to determine rhizobacterial composition^27^. Natural variation of the rhizodeposits could be influencing bacterial assemblages in the three rhizobacterial communities analyzed^88^. Other factors such as root system architecture would also probably be involved in the differences observed among grasses and non-grass plants^88^. Nevertheless, there may be other factors rather than plant genotype potentially affecting the community structure. For example, the microenvironment generated by a whole set of plants^19^ or bacterial interactions (competition and/or cooperation)^62^.

Finally, we also detected intraspecific variability (among replicates), which could be a consequence of working with natural samples in uncontrolled environmental conditions. For instance, differences in the stages of plant development or plant age are factors that could affect the rhizosphere microbiome assemblage^14,18,24,89–91^ and could be the cause of some of the observed variability. Also, in the rhizosphere, the intraspecific variability could be a consequence of weak or non-stable relationships^87^.

In summary, based on our results we conclude that the diversity and structure of rhizobacterial communities of native plants in the Chilean altiplanic grasslands are affected by a combination of biotic and abiotic factors. Thus, native plants might recruit and conserve specific growth-promoting bacteria, allowing them to survive in one of the harshest environments on Earth. Nevertheless, we are aware of the limitations of our study due to the small sample size, which makes that some of the results could not be explained with confidence. We believe that further and more exhaustive studies are needed to address the environmental factors that drive the structure of the rhizosphere to understand the role of specific groups of bacteria in promoting plant growth, which is particularly important considering the extremely adverse climate and poor nutrient soils of this particular environment. We also propose that the RSS might be a soil fraction that introduces restrictions and/or promotes the recruitment of a subset of bacteria that colonize the rhizosphere from the surrounding bulk soil, but to validate this statement further studies are also required. Meanwhile, this work sets a baseline for soil microbiome studies in the higher parts of the Atacama Desert considering three soil compartments and offers interesting data for comparison with other (extreme or not) environments around the globe.

## Methods

### Sampling area and sample collection

Sampling was carried out during the austral summer (April 2016) in an area located between 3,870 and 4,270 m.a.s.l., in the Atacama Desert, western Andes (~23.5°S), corresponding to the Andean steppe grasslands^46^. The soils in this area are classified as Aridisols. In order to indicate the precise place where the plants were sampled, we assigned three sites, which corresponded to sites 3, 4, and 7 (Fig. 1a,b) of the Talabre-Lejía Transect (TLT; 20 sites in total) previously described^46,62^. The plant species chosen for the study, especially the three perennial grasses *Calamagrostis crispa* (site 3), *Nassella nardoides* (site 3), and *Jarava frigida* (site 7) (Fig. 1e–g) showed the highest plant coverage in all three sites. Additionally, we included a non-grass plant, *Pycnophyllum bryoides* (site 4) (Fig. 1h) also native and representative of the Altiplano for comparative purposes. From a methodological point of view, grasses develop an extensive fibrous root system, forming a mesh that maintains an adequate amount of soil closely associated to the roots. This feature enabled us to sample the entire root system with the minimum of variability among plants. Three different individuals of each plant (triplicates) were selected giving a total of 36 samples. Three different soil compartments were sampled according to their distance to the roots: the rhizosphere (bacteria firmly attached to the roots), the rhizosphere-surrounding soil (RSS; bacteria loosely attached to the roots), and the bulk soil (plant-free soil).

For each plant, the whole root system was placed in sterile plastic bags and stored at 4 °C. The root system was gently shaken and the particles detached during shaking were defined as the RSS. A total of approximately 100 g of RSS for each plant was obtained. The rhizosphere was the soil detached, after the roots were washed in a 10 mM NaCl solution^65,92^ and collected in 50 ml tubes. For sampling the bulk soil, around 100 g of soil without the presence of any growing plants (at least 1 m away from each sampled plant) were recovered in sterility, after removing the first layer of altered soil. Bulk soil samples were extracted at 15 cm deep in the case of the Poaceae plants that had longer roots and at 10 cm deep in the case of the Caryophyllaceae with shorter roots. All soils were immediately stored in dry ice until their arrival at the laboratory where they were frozen at −80 °C.

### Environmental and soil physicochemical measurements

A portion of the bulk soil samples was used to determine the metal composition using Total Reflection X-Ray Fluorescence (TXRF) technique. Briefly, for each sample 1 g of soil was resuspended in 1 mL of distilled water and homogenized for 2 h at room temperature. After mixing, the samples were centrifuged at 11,440 g for 10 min in a Hettich Universal 32R. The soluble fraction was recovered and measured in a Bruker S2 PICOFOX.

Soil texture (sand:silt:clay), electrical conductivity, and nitrogen levels (total N, NO_3_^−^, NH_4_^+^) were provided by the Laboratorio Agroanálisis UC, Facultad de. Agronomía e Ingeniería Forestal, Pontificia Universidad Católica de Chile, according to the methods established by the Normalization and Accreditation Commission (CNA) of the Chilean Society of Soil Science^93^, as performed before^62^.

For soil pH measurement, 2 g of soil were mixed with 5 mL milliQ water and homogenized during 2 h at RT (S1000 Gyrotwister) at 60 rpm. Afterwards, the soil was decanted during 2 h. The pH was measured in triplicate for each sample in a Thermo Scientific Orion 3 star benchtop pH meter.

### DNA extraction, PCR amplification, and high-throughput sequencing

Total DNA from rhizosphere, RSS and bulk soils was extracted from each sample (10 g each) using a modification of NucleoSpin® Food kit (Macherey-Nagel), following the manufacturer’s instructions. The lysis buffer of that kit that is more suitable for food DNA extraction was substituted for the following buffer: 100 mM Tris-HCl; pH 8, 100 mM Na EDTA; pH 8, 100 mM Na_2_HPO_4_, 1.5 M NaCl, 1% (w/v) CTAB. Concentration and purity were quantified using Infinite® 200 PRO (Tecan®) reader. Microbial 16S rRNA gene was amplified using the bacteria-specific primer set 28 F (5′ GA GTT TGA TCM TGG CTC AG 3′) and 519R (5′ GWA TTA CCG CGG CKG CTG 3′), flanking variable regions V1–V3 of the 16S rRNA gene^94^, with a barcode in the forward primer. For the amplification, the kit HotStarTaq Plus Master Mix (Qiagen) was used with the following conditions: 94 °C 3 min, 28 cycles of 94 °C 3 s, 53 °C 4 s, and 72 °C 1 min, followed by an elongation phase of 72 °C 5 min. PCR products were examined with an agarose gel (2%). Samples were purified using Agencourt AMPure XP (Beckman Coulter, Inc; Indianapolis, USA). DNA libraries were constructed following the protocol TruSeq DNA sample preparation (Illumina, Inc; San Diego, USA). Sequencing was performed by MrDNA Next Generation Sequencing Service Provider (Shallowater, Texas, USA) on Illumina MiSeq platform in an overlapping 2 × 300 bp configuration to obtain a minimum throughput of 40,000 sequences (reads) per sample.

### Sequence analysis and taxonomical assignation

The 16S rRNA amplicons were processed and analyzed following previously described protocols^95,96^. Briefly, reads were overlapped by pairs and cleaned of barcode. Sequences <150 bp or with ambiguous assignation were discarded. Valid sequences were grouped using USearch (v6.1.544) with 4% of divergence in order to remove chimeras and singletons^97,98^. Finally, sequences were filtered with a minimum quality of 30 (q30) with Mothur v1.22.2^99^. Taxonomical assignation was done using the software Quantitative Insights Into Microbial Ecology, QIIME v1.8.0^100^. Operational Taxonomical Units (OTUs) were identified at 97% identity against GreenGenes r16S database^101^ with USearch v6.1.544^97,98^ using default parameters in QIIME. An OTU was considered valid if it was present in at least one of the three replicates. The OTUs with mitochondrial or chloroplast assignation were removed. The OTUs identification numbers, abundance and taxonomy retrieved from GreenGenes database for all samples are specified in Table S1.

### Accession numbers

All 16S rRNA gene sequence data used in this study is deposited in the Sequence Read Archive (SRA) of the National Center for Biotechnology Information (NCBI) under the BioProject accession number PRJNA416778.

### Statistical analysis

To perform alpha-diversity analyses, each sample was randomly subsampled (without replacement) using the alpha_rarefaction.py script found in QIIME^100^ to generate Shannon, Chao1, and evenness (Shannon’s equitability index) indices along with the observed number of OTUs at different sampling depths. Rarefaction curves for each of these metrics were obtained by serial subsampling (in increments of 1,600 sequences and 10 iterations per increment) to a standardized depth of 16,000 sequences per sample. For the multivariate analyses we used the software R v.3.1.3^102^.

Differences in community composition and PCoA were calculated using unweighted UniFrac metrics^103^, with rarefied communities, using QIIME internal scripts.

Shapiro-Wilk normality test (p < 0.05) was performed to check the distribution of our samples, which resulted to be not normally distributed. Non-parametric Kruskal-Wallis test of variance (p < 0.05) was performed for comparing the alpha-diversity among compartments. Post-hoc comparisons were performed using Dunn’s test. All these analyses were done using R software with package *stats*.

Pairwise statistical analyses were performed using Mann-Whitney U test (p < 0.05). The resulting p-values were adjusted using Benjamini-Hochberg as a FDR correction method.

To identify OTU enrichment in each of the three compartments, we employed a linear statistics model on relative abundance value (log2; >0.03% threshold) using a variation of R script (package *limma*) previously reported^104^. Differentially abundant OTUs between compartments were calculated using Bayes moderated t-test^105^. The resulting p-values were adjusted for multiple hypotheses testing using Benjamini-Hochberg as FDR correction method. Ternary plots were performed using *ternaryplot* function of R package *vcd*.

## Supplementary information

Supplementary Material

Supplementary Material Table S2
