# Supplementary material for: Bacterial communities associated to Chilean altiplanic native plants from the Andean grasslands soils
Source: Sci Rep. 2019 Jan 31;9:1042. doi: 10.1038/s41598-018-37776-0 (PMC6355873; doi:10.1038/s41598-018-37776-0)
Supplement: Supplementary file 1 — Supplementary Material [file 41598_2018_37776_MOESM1_ESM.docx]

**Bacterial communities associated to Chilean altiplanic native plants from the Andean grasslands soils**

Beatriz Fernández-Gómez^1,2^ , Jonathan Maldonado^1,2^, Dinka Mandakovic^1,2^, Alexis Gaete^1,2^, Rodrigo A. Gutiérrez^1,3,4^, Alejandro Maass^1,5,6^, Verónica Cambiazo^1,2^ & Mauricio González^1,2^

^1^FONDAP Center for Genome Regulation, Santiago, Chile

^2^ Department of Bioinformatics and Genetic Expression, Institute of Nutrition and Food Technology, Universidad de Chile, El Libano 5524, Macul, Santiago, Chile

^3^Department of Molecular Genetics and Microbiology, Pontificia Universidad Católica de Chile, Av. Libertador Bdo O’Higgins 340, Santiago, Chile

^4^Millennium Institute for Integrative Plant Systems and Synthetic Biology, Pontificia Universidad Católica de Chile, Av. Libertador Bernardo O’Higgins 340, Santiago, Chile

^5^Mathomics, Center for Mathematical Modeling, Universidad de Chile, Beauchef 850, Santiago, Chile

^6^Department of Mathematical Engineering, Universidad de Chile, Beauchef 850, Santiago, Chile

Correspondence and requests for materials should be addressed to M.G. (email: mgonzale@inta.uchile.cl)

**SUPPLEMENTARY INFORMATION**

**Supplementary Tables**

| **Characteristic** | **Site 3** | **Site 4** | **Site 7** | **3 vs 4** | **3 vs 7** | **4 vs 7** |
| --- | --- | --- | --- | --- | --- | --- |
| **Altitude (m.a.s.l.)** | 4,270 | 4,170 | 3,870 | - | - | - |
| **-Lat; -Long** | 23.43; 67.77 | 23.42; 67.78 | 23.39; 67.01 | - | - | - |
| **pH** | 5.8 ±0.064 | 5.3 ±0.208 | 5.5 ±0.152 | n.s | n.s | n.s |
| **EC (mS/cm)** | 0.034 ±0.005 | 0.091 ±0.065 | 0.073 ±0.045 | n.s | n.s | n.s |
| **Sand (%)** | 86.2 ±4.450 | 85.4 ±3.104 | 87.02 ±4.916 | n.s | n.s | n.s |
| **Silt (%)** | 9.9 ±1.458 | 10.34 ±3.393 | 9.29 ±2.983 | n.s | n.s | n.s |
| **Clay (%)** | 0.66 ±0.082 | 1.08 ±0.159 | 1.67 ±0.889 | n.s | n.s | n.s |
| **OM (%)** | 1.412 ±0.359 | 4.672 ±3.604 | 2.346 ±0.809 | n.s | n.s | n.s |
| **As (mg/kg)** | 0.012 ±0.004 | 0.012 ±0.002 | 0.009 ±0.001 | n.s | * | n.s |
| **Ba (mg/kg)** | 0.072 ±0.055 | N.D | 0.006 ±0.000 | N.D | n.s | N.D |
| **Br (mg/kg)** | 0.09 ±0.057 | 0.188±0.11 | 0.083 ±0.011 | n.s | n.s | n.s |
| **Ca (mg/kg)** | 4.158 ±1.271 | 3.329 ±0.423 | 5.9 ±1.015 | n.s | * | n.s |
| **Cu (mg/kg)** | 0.02 ±0.011 | 0.029 ±0.015 | 0.014 ±0.003 | n.s | n.s | n.s |
| **Fe (mg/kg)** | 3.889 ±2.519 | 1.45 ±0.215 | 2.013 ±0.604 | n.s | * | n.s |
| **N (mg/kg)** | 8.917 ±0.879 | 11.642 ±2.909 | 10.033 ±3.001 | n.s | n.s | n.s |
| **N (%)** | 0.035 ±0.013 | 0.052 ±0.030 | 0.058 ±0.023 | n.s | n.s | n.s |
| **NH_4_ (mg/kg)** | 6.163 ±2.751 | 8.25 ±1.393 | 7.814 ±2.768 | n.s | n.s | n.s |
| **NO_3_ (mg/kg)** | 3.088 ±1.661 | 3.725 ±4.659 | 2.886 ±0.834 | n.s | n.s | n.s |
| **K (mg/kg)** | 5.334 ±1.220 | 5.363 ±0.584 | 2.389 ±2.873 | n.s | * | n.s |
| **Na (mg/kg)** | 1.615 ±0.185 | 3.502 ±0.105 | 3.509 ±0.000 | n.s | n.s | n.s |
| **P (mg/kg)** | 0.521 ±0.379 | 0.339 ±0.107 | 0.399 ±0.179 | n.s | * | n.s |
| **Zn (mg/kg)** | 0.106 ±0.089 | 0.012 ±0.002 | 0.09 ±0.106 | n.s | n.s | n.s |

**Supplementary Table S1**. **Geographic location, soil properties, and site comparison (p-value)**. Values in columns 2, 3, and 4 represent the mean among the three replicates ± standard deviation. Values in columns 5, 6, and 7 show the significant differences (p-value) between site pairs (site 3 versus site 4; site 3 versus site 7; and site 4 versus site 7). EC: Electric conductivity; OM: organic matter; As: arsenic; Ba: barium; Br: bromine; Ca: calcium; Cu: copper Fe: iron; N: nitrogen; NO_3_: nitrate; NH_4_: ammonium; K: potassium; Na: sodium P: phosphorus; Zn: zinc. N.D: not determined; n.s: not significant; * p < 0.05 (Mann-Whitney U test; FDR adjusted p-value<0.05).

**Supplementary Table S2**. List of all OTUs and their taxonomy assigned by GreenGenes r16S database for all samples (excel file).

| **Plant** | **Compartment** | **Shannon’s** | **Chao1** | **Evenness** |
| --- | --- | --- | --- | --- |
| CAL R1 | Bulk soil | 8.81 | 4138.00 | 0.79 |
| CAL R2 | Bulk soil | 9.41 | 4075.52 | 0.83 |
| CAL R3 | Bulk soil | 8.84 | 3207.02 | 0.80 |
| NAS R1 | Bulk soil | 9.53 | 4066.08 | 0.84 |
| NAS R2 | Bulk soil | 8.69 | 2784.06 | 0.80 |
| NAS R3 | Bulk soil | 8.57 | 3499.17 | 0.78 |
| JAR R1 | Bulk soil | 8.30 | 3202.61 | 0.76 |
| JAR R2 | Bulk soil | 8.61 | 3443.81 | 0.78 |
| JAR R3 | Bulk soil | 9.73 | 3868.77 | 0.86 |
| PYC R1 | Bulk soil | 9.21 | 3374.48 | 0.83 |
| PYC R2 | Bulk soil | 8.56 | 3710.14 | 0.77 |
| PYC R3 | Bulk soil | 8.11 | 2939.90 | 0.75 |
| CAL R1 | RSS | 9.50 | 4530.02 | 0.84 |
| CAL R2 | RSS | 8.95 | 4335.90 | 0.80 |
| CAL R3 | RSS | 8.75 | 3982.92 | 0.78 |
| NAS R1 | RSS | 9.43 | 4148.88 | 0.83 |
| NAS R2 | RSS | 9.49 | 3200.13 | 0.86 |
| NAS R3 | RSS | 9.15 | 3797.15 | 0.82 |
| JAR R1 | RSS | 4.79 | 2328.20 | 0.47 |
| JAR R2 | RSS | 9.46 | 3881.67 | 0.84 |
| JAR R3 | RSS | 9.10 | 3452.38 | 0.82 |
| PYC R1 | RSS | 8.67 | 3624.14 | 0.79 |
| PYC R2 | RSS | 8.60 | 3298.61 | 0.78 |
| PYC R3 | RSS | 9.32 | 3303.35 | 0.84 |
| CAL R1 | Rhizosphere | 8.38 | 2936.17 | 0.78 |
| CAL R2 | Rhizosphere | 6.12 | 2557.11 | 0.58 |
| CAL R3 | Rhizosphere | 8.54 | 3207.92 | 0.78 |
| NAS R1 | Rhizosphere | 8.28 | 2905.49 | 0.77 |
| NAS R2 | Rhizosphere | 7.70 | 2996.15 | 0.72 |
| NAS R3 | Rhizosphere | 8.46 | 3241.50 | 0.78 |
| JAR R1 | Rhizosphere | 7.00 | 2601.98 | 0.67 |
| JAR R2 | Rhizosphere | 5.57 | 1822.71 | 0.56 |
| JAR R3 | Rhizosphere | 7.85 | 3082.20 | 0.73 |
| PYC R1 | Rhizosphere | 6.88 | 1774.72 | 0.70 |
| PYC R2 | Rhizosphere | 6.64 | 1943.90 | 0.66 |
| PYC R3 | Rhizosphere | 8.19 | 2685.34 | 0.76 |

**Supplementary Table S3. Indexes of diversity (Shannon’s), richness (Chao1), and evenness for each sample.** CAL: *Calamagrostis crispa;* NAS: *Nassella nardoides;* JAR: *Jarava frigida;* PYC: *Pycnophyllum bryoides*. RSS: rhizosphere-surrounding soil. Sample replicates are marked as R1, R2, and R3.

|  | **Shannon** | | **Chao1** | **Evenness** |
| --- | --- | --- | --- | --- |
| **Rhizosphere-** **RSS** | | *** | *** | *** |
| **Rhizosphere-bulk soil** | | ** | ** | ** |
| **RSS -bulk soil** | | n.s | n.s | n.s |

**Supplementary Table S4. Pairwise Dunn’s test (adjusted p-value <0.05) for the three indices of alpha-diversity.** Significance level: n.s: non-significant; * p < 0.05, ** p < 0.01, *** p < 0.001. RSS: rhizosphere-surrounding soil.

| **Poaceae** | **R vs. RSS** | **R vs. BS** | **RSS vs. BS** |
| --- | --- | --- | --- |
| Acidobacteria-6 | * | *** | n.s |
| Acidobacteriia | n.s | ** | n.s |
| Actinobacteria | n.s | * | n.s |
| Alphaproteobacteria | n.s | ** | n.s |
| Bacilli | n.s | n.s | n.s |
| Betaproteobacteria | n.s | n.s | n.s |
| Blastocatellia | n.s | ** | n.s |
| Cytophagia | n.s | n.s | n.s |
| Deltaproteobacteria | * | ** | n.s |
| Flavobacteriia | n.s | n.s | n.s |
| Gammaproteobacteria | n.s | n.s | n.s |
| Gemmatimonadetes | * | *** | n.s |
| iii1-8 | * | *** | n.s |
| Opitutae | ** | *** | n.s |
| Planctomycetia | n.s | ** | n.s |
| Saprospiria | n.s | n.s | n.s |
| Spartobacteria | n.s | n.s | n.s |
| Sphingobacteriia | n.s | * | n.s |
| Thermoleophilia | * | *** | n.s |
| **Caryophyllaceae** |  |  |  |
| Acidobacteria-6 | n.s | n.s | n.s |
| Acidobacteriia | n.s | n.s | n.s |
| Actinobacteria | n.s | n.s | n.s |
| Alphaproteobacteria | n.s | n.s | n.s |
| Anaerolineae | n.s | n.s | n.s |
| Betaproteobacteria | n.s | n.s | n.s |
| Blastocatellia | n.s | n.s | n.s |
| Deltaproteobacteria | n.s | n.s | n.s |
| Fimbriimonadia | n.s | n.s | n.s |
| Gammaproteobacteria | n.s | n.s | n.s |
| Gemmatimonadetes | n.s | n.s | n.s |
| iii1-8 | n.s | n.s | n.s |
| Opitutae | n.s | n.s | n.s |
| Planctomycetia | n.s | n.s | n.s |
| Saprospiria | n.s | n.s | n.s |
| SJA-28 | n.s | n.s | n.s |
| Spartobacteria | n.s | n.s | n.s |
| Sphingobacteriia | n.s | n.s | n.s |
| Thermoleophilia | n.s | n.s | n.s |
| TM7-3 | n.s | n.s | n.s |
| vadinHA49 | n.s | n.s | n.s |

**Supplementary Table S5. Significant differences (Mann-Whitney U test; FDR adjusted p-value <0.05) in taxa abundances (class level) between compartments**. Significance level: n.s: non-significant; * p < 0.05, ** p < 0.01, *** p < 0.001. BS: bulk soil; RSS: rhizosphere-surrounding soil; R: rhizosphere.

|  | **CAL** | | **NAS** | | **JAR** | |
| --- | --- | --- | --- | --- | --- | --- |
| **Phylum/class** | **Enriched** | **Depleted** | **Enriched** | **Depleted** | **Enriched** | **Depleted** |
| Acidobacteria | 6 | 44 | 26 | 148 | 9 | 21 |
| Actinobacteria | 4 | 5 | 14 | 37 | 4 | 9 |
| AD3 | 0 | 0 | 0 | 0 | 1 | 0 |
| Armatimonadetes | 0 | 0 | 0 | 1 | 0 | 0 |
| Bacteroidetes | 4 | 10 | 24 | 9 | 4 | 12 |
| Chloroflexi | 0 | 3 | 0 | 8 | 0 | 0 |
| Elusimicrobia | 0 | 0 | 0 | 1 | 0 | 1 |
| FBP | 0 | 0 | 4 | 0 | 0 | 1 |
| Fibrobacteres | 0 | 0 | 0 | 1 | 0 | 0 |
| Firmicutes | 0 | 0 | 0 | 0 | 1 | 0 |
| Gemmatimonadetes | 0 | 6 | 0 | 13 | 1 | 3 |
| Nitrospirae | 0 | 0 | 0 | 3 | 1 | 2 |
| Planctomycetes | 2 | 6 | 2 | 9 | 0 | 5 |
| Proteobacteria | 23 | 15 | 104 | 40 | 12 | 60 |
| α-Proteobacteria | 16 | 2 | 69 | 13 | 8 | 41 |
| β-Proteobacteria | 3 | 9 | 28 | 17 | 0 | 10 |
| δ-Proteobacteria | 2 | 0 | 0 | 7 | 1 | 1 |
| γ-Proteobacteria | 2 | 3 | 7 | 3 | 3 | 8 |
| TM6 | 0 | 0 | 0 | 0 | 0 | 1 |
| TM7 | 0 | 0 | 0 | 1 | 0 | 1 |
| Verrucomicrobia | 2 | 8 | 5 | 10 | 0 | 2 |
| **Total** | **41** | **97** | **179** | **281** | **33** | **118** |

**Supplementary Table S6. Number of OTUs significantly enriched or depleted in the rhizosphere for each plant species at phylum level.** Phylum Proteobacteria was also analyzed at class level.

| **OTU id** | **Phylum** | **Class** | **Order** | **Family** | **Genus** |
| --- | --- | --- | --- | --- | --- |
| **CAL** |  |  |  |  |  |
| 71588 | Proteobacteria | Alphaproteobacteria | Sphingomonadales | Sphingomonadaceae | *Sphingomonas* |
| 4410018 | Proteobacteria | Deltaproteobacteria | Desulfuromonadales | Geobacteraceae | *Geobacter* |
| 4418692 | Proteobacteria | Deltaproteobacteria | Desulfuromonadales | Pelobacteraceae | *-* |
| **NAS** |  |  |  |  |  |
| 261726 | Actinobacteria | Actinobacteria | Actinomycetales | Actinosynnemataceae | *Lentzea* |
| 4451387 | Actinobacteria | Actinobacteria | Actinomycetales | Microbacteriaceae | *Mycetocola* |
| 277766 | Bacteroidetes | Flavobacteriia | Flavobacteriales | Weeksellaceae | *Chryseobacterium* |
| 1085831 | Bacteroidetes | Sphingobacteriia | Sphingobacteriales | Sphingobacteriaceae | *Pedobacter* |
| 1142725 | Bacteroidetes | Sphingobacteriia | Sphingobacteriales | Sphingobacteriaceae | *Pedobacter* |
| 220406 | Bacteroidetes | Flavobacteriia | Flavobacteriales | Weeksellaceae | *Chryseobacterium* |
| 2952992 | Bacteroidetes | Sphingobacteriia | Sphingobacteriales | Sphingobacteriaceae | *Pedobacter* |
| 229949 | Bacteroidetes | Flavobacteriia | Flavobacteriales | Weeksellaceae | *Chryseobacterium* |
| 1097562 | Bacteroidetes | Sphingobacteriia | Sphingobacteriales | Sphingobacteriaceae | *-* |
| 264030 | Bacteroidetes | Flavobacteriia | Flavobacteriales | Weeksellaceae | *Chryseobacterium* |
| 1115401 | Bacteroidetes | Flavobacteriia | Flavobacteriales | Weeksellaceae | *Chryseobacterium* |
| 1103943 | FBP | - | - | - | - |
| 985421 | Planctomycetes | Phycisphaerae | WD2101 | - | *-* |
| 4338095 | Proteobacteria | Alphaproteobacteria | Rhodospirillales | Acetobacteraceae | *Roseomonas* |
| 808323 | Proteobacteria | Alphaproteobacteria | Sphingomonadales | Sphingomonadaceae | *Kaistobacter* |
| 4451370 | Proteobacteria | Gammaproteobacteria | Pseudomonadales | Pseudomonadaceae | *Pseudomonas* |
| 4363161 | Proteobacteria | Alphaproteobacteria | Sphingomonadales | Sphingomonadaceae | *Kaistobacter* |
| 225881 | Proteobacteria | Alphaproteobacteria | Sphingomonadales | Sphingomonadaceae | *Kaistobacter* |
| 1939291 | Proteobacteria | Gammaproteobacteria | Pseudomonadales | Pseudomonadaceae | *Pseudomonas* |
| 88480 | Proteobacteria | Alphaproteobacteria | Rhizobiales | Bradyrhizobiaceae | *Bosea* |
| 25807 | Proteobacteria | Alphaproteobacteria | Sphingomonadales | Sphingomonadaceae | *-* |
| 225220 | Proteobacteria | Alphaproteobacteria | Rhizobiales | Bradyrhizobiaceae | *Bosea* |
| 4469179 | Proteobacteria | Gammaproteobacteria | Pseudomonadales | Pseudomonadaceae | *Pseudomonas* |
| **JAR** |  |  |  |  |  |
| 3645757 | Acidobacteria | Blastocatellia | RB41 | - | *-* |
| 4483956 | Acidobacteria | Blastocatellia | RB41 | - | *-* |
| 4479507 | Actinobacteria | Actinobacteria | Actinomycetales | - | *-* |
| 292178 | Proteobacteria | Alphaproteobacteria | Rhizobiales | Bradyrhizobiaceae | *Bosea* |
| 4317750 | Proteobacteria | Deltaproteobacteria | Myxococcales | Haliangiaceae | *-* |
| 676387 | Proteobacteria | Alphaproteobacteria | Rhizobiales | Methylocystaceae | *-* |
| 888801 | Proteobacteria | Gammaproteobacteria | Pseudomonadales | Pseudomonadaceae | *Pseudomonas* |

**Supplementary Table S7. Taxonomy of the enriched and exclusive OTUs found in the rhizosphere for each plant species.**

**SUPPLEMENTARY FIGURES**

**Supplementary Figure S1. Venn diagrams showing, for each plant species, the number of shared (core microbiome) and exclusive (non-core) OTUs among the three compartments.** CAL: *Calamagrostis crispa;* NAS: *Nassella nardoides;* JAR: *Jarava frigida;* PYC: *Pycnophyllum bryoides.* RSS: rhizosphere-surrounding soil.

**Supplementary Figure S2. (a) Relative abundance for each phylum (>1% in at least one sample) in the three compartments of *Pycnophyllum bryoides* (PYC). (b) Table showing the actual percentages.** Numbers in brackets represent the standard deviation. RSS: rhizosphere-surrounding soil.

**Supplementary Figure S3. Taxonomy and relative abundance for each plant species and compartment at class level (>1% in at least one sample).** CAL: *Calamagrostis crispa;* NAS: *Nassella nardoides;* JAR: *Jarava frigida;* PYC: *Pycnophyllum bryoides.* RSS: rhizosphere-surrounding soil.

**Supplementary Figure S4.** Venn diagrams for **(a)** rhizosphere enriched OTUs **(b)** rhizosphere depleted OTUs, and **(c)** rhizosphere enriched and exclusive OTUs for all plant species (except for PYC that did not show any enrichment or depletion).
